# Pain as a risk factor of depression and anxiety symptoms with multiple myeloma during maintenance therapy

**DOI:** 10.3389/fpsyg.2022.1015497

**Published:** 2022-11-30

**Authors:** Hui Shi, Honglin Ren, Ying Tian, Zhe Chen, Cuiping Xu, Lu Lu, Xiaoyu Ma, Xiaoyan Li, Wenming Chen, Tong Guo, Cuizhen Fan, Wen Gao

**Affiliations:** ^1^Department of Clinical Psychology, Beijing Chao-Yang Hospital, Capital Medical University, Beijing, China; ^2^Department of General Education, Wuhan Vocational College of Software and Engineering, Wuhan, China; ^3^Department of Hematology, Myeloma Research Center of Beijing, Beijing Chao-Yang Hospital, Capital Medical University, Beijing, China; ^4^Department of Endocrinology, Beijing Chaoyang Hospital, Capital Medical University, Beijing, China; ^5^Department of Respiratory and Critical Care Medicine, Beijing Bo-Ai Hospital, China Rehabilitation Research Center, Beijing, China; ^6^Department of Cardiology, Beijing Huai-Rou Hospital, Beijing, China; ^7^Clinical Laboratory, Tai-Yang-Gong Community Health Service Center, Beijing, China; ^8^Department of Hematology, Bayannaoer Hospital, Inner Mongolia, China; ^9^The National Clinical Research Center for Mental Disorders and Beijing Key Laboratory of Mental Disorders, Beijing Anding Hospital, Beijing, China; ^10^Department of Oncology, Beijing Chao-Yang Hospital, Capital Medical University, Beijing, China

**Keywords:** myeloma, depression, anxiety disorders, mood disorders, cancer pain

## Abstract

**Objective:**

To investigate the prevalence of depression or anxiety in patient with multiple myeloma (MM) in China during maintenance treatment and its associated influencing factors.

**Methods:**

Patients with MM (*n* = 160) received maintenance therapy, and control subjects (without MM, *n* = 160) matched on age, sex, and BMI were recruited. Patients completed questionnaires, including the Patient Health Questionnaire-9 (PHQ-9), the Generalized Anxiety Disorder 7-item Scale (GAD-7), and the Verbal Pain Rating Scale (VPRS). Data on the Clinical characteristics, biochemical indicators of *de novo* MM were from the database of the Hematology Department of Beijing Chao-yang Hospital. Multiple linear regression model analysis was used to compare the differences in PHQ-9 and GAD-7 scale scores between the control group and the case group after correction for relevant variables. Multiple logistic regression models were subsequently used to analyze the correlation between the presence or absence of anxiety and depression and clinical indicators in the MM groups.

**Results:**

Depression symptoms was present in 33.33% and anxiety symptoms in 24.68% of first-episode MM in the maintenance phase of treatment, and depression symptoms in the index-corrected MM group was significantly different from that in the control group (*t* = 2.54, *P* < 0.05). Analyses of multiple logistic regressions: biochemical indicators and clinical typing were not significantly associated with anxiety and depression. Compared to the pain rating 1, the risk of depressive mood was greater in the case group with the pain rating 2 (OR = 2.38) and the pain rating ≥ 3 (OR = 4.32). The risk of anxiety was greater in the case group with the pain rating ≥ 3 than the pain rating 1 (OR = 2.89).

**Conclusion:**

Despite being in clinical remission, depressive mood problems in patients with MM remain prominent. Clinicians should enhance mood assessment and management in patients with concomitant pain.

## Introduction

Multiple myeloma (MM), a plasma cell malignancy, has the second-highest prevalence among hematological malignancies, accounting for 1% of all cancers and 15% of hematologic neoplasms (Michels and Petersen, 2017). In a study on MM in China, the standardized incidence rate was 0.9 per 100,000 person-years when standardized by 2000 China population census data (Gao et al., 2022). The treatment of MM typically comprises multiple phases. For transplant-eligible patients, treatments include induction therapy and autologous stem cell transplantation (ASCT), followed by consolidation and maintenance therapy. Maintenance therapy refers to treatment that differs from previous, more intensive therapy, typically single-agent or doublet therapy following ASCT (Dimopoulos et al., 2020). For transplant non-eligible patients, maintenance therapy is most widely used after induction treatment followed by consolidation. With improvements in survival, maintenance therapy has become a longer-term of medication administered to prevent disease progression and/or relapse (Turesson et al., 2018).

Recent research has revealed that depression and anxiety in cancer patients are strictly related to poor treatment compliance, low quality of life, and reduced survival rate (Lee et al., 2017). The prevalence rates of diagnosed depression disorder and anxiety disorder were 12.0% and 8.6%, respectively, among general hospital outpatients in China (He et al., 2009). The prevalence of anxiety and depression is up to 20% and 10%, respectively, among patients with cancer during the active and palliative treatment periods, which is higher than that in the general population (Mitchell et al., 2011). Although it is crucial, these issues are often given little attention by both doctors and patients. 30–40% of cancer patients experience psychological problems that go undetected by clinicians (Pitman et al., 2018). Depression and anxiety vary among tumor types due to tumor-related neurological effects and treatment-related side effects. Depression and anxiety increased risk of lung, mouth, prostate, and skin cancers (Wang et al., 2020). Also, anxiety often accompanies depression among patients with all types of cancer.

MM is one of the common diseases in human hematological system. Patients with hematological malignancies tumors generally have more complications than patients with solid tumors (Rolston, 2017). Hematological tumors are systemic and related to bone marrow. Hematological malignancies are more likely to require high dose chemotherapy and undergo allo or auto-SCT. Approximately 17% of hematological cancer patients have suffered clinical anxiety and/or depression during treatment with diagnosis (Clinton-McHarg et al., 2014; Posluszny et al., 2019). Many studies have focused on mixed groups of hematological cancer survivors rather than specific survivors (Hall et al., 2013a,b, 2014; El-Jawahri et al., 2020). The prevalence of anxiety or depression varies among hematological malignancies (Oberoi et al., 2017). Substantial proportions of patients with MM reported psychosocial wellbeing in ranges suggestive of impairment with respect to depression (17%) and anxiety (20%) (Zaleta et al., 2020). In some studies, anxiety, and depression are higher in the pre-ASCT period than in the post-ASCT period, which are associated with psychological distress. However, other studies on this issue have been inconsistent (Chakraborty et al., 2018). Notably, there is one possible reason for these conflicting findings—most patients are grouped by disease progression instead of final and definitive clinical stages. Patients in the maintenance stage still experience psychological disorders that are often overlooked by doctors, despite the remission of pathological symptoms. Exploring the relationship between hematological tumors and depression is helpful to explore the deep biological causes of depression.

There are two potential causes for depression and anxiety in patients with cancer. In essence, one is the biopsychosocial model, and the other is the specific neuropsychiatric effects of certain cancers and its associated treatments (Pitman et al., 2018). Levels of the cytokine interleukin-6 and ectopic parathormone-related peptide production may be relevant in the pathological process of MM (Bortolato et al., 2017). It becomes evident that baseline clinical symptoms, such as anemia or osteolytic lesion, had become poorly predictive. We hypothesize that MM patients’ anxiety and depression during the period of maintenance therapy, their diagnosis, and treatment modalities are interrelated. On the other hand, pain is one of the most common somatic symptoms with MM during maintenance therapy (Xu et al., 2019; Ludwig et al., 2020). Because of pain and depression sharing some parts of common neural pathways, it is widely believed that pain has been identified as a risk factor for depression. Few studies proved that the relationship between symptoms of depression, anxiety, and pain was well established in MM. If the intensity of pain increases the prevalence of emotional symptoms in MM, our study may serve as an essential part of systematic interventions to mood disturbance.

Given the adverse effects of depression and anxiety in patients with malignant tumors, it is crucial to identify them clinically. Previous studies have examined the relationship between anxiety, depression and myeloma. Five years after the diagnosis of myeloma patients, about 27.4% of patients reported signs of anxiety and 25.2% reported signs of depression (Molassiotis et al., 2011; Lamers et al., 2013; Copeland et al., 2017). However, the sample size of most studies was small, and the stress reactions in the early stage of treatment were not distinguished. In addition, no researches have shown their associations and the risks of manifesting the characteristics of *de novo* MM during the maintenance period. To our knowledge, no previous studies have analyzed the prevalence of anxiety and depression symptoms among Chinese MM patients with maintenance therapy. The current study tries to fill a gap in the literature to help doctors identify the signs of anxiety and depression to prevent its progression using the interventional treatment. In this study, 160 patients with MM were selected as big data samples to explore the risk factors of depression and anxiety during maintenance therapy. It is not only helpful to identify and diagnose them, but also has value for their clinical treatment. The major aims of this study, therefore, is to investigate the relationship between biological indicators of MM and depression or anxiety, and to explore whether depression or anxiety are disease-specific changes in MM.

## Methods and procedure

### Participates and study setting

From May 2017 to April 2019, outpatients with MM who received maintenance therapy in Beijing Chao Yang Hospital were included in this case-control study. Patients were included, if they: (1) were diagnosed according to the International Myeloma Working Group (IMWG) 2006 diagnosis criteria (Durie et al., 2006), in the period of maintenance therapy after treatment, and (2) aged between 30 and 80 years old, and (3) were proficient in reading and writing Mandarin, and (4) signed informed consent form. Participants were excluded, if they: (1) were diagnosed with serious physical diseases such as cerebrovascular disease, liver and kidney disease, or endocrine system disease; and (2) past medical history has been diagnosed with severe mental illness (such as schizophrenia) requiring antipsychotic medications. The control group was the healthy community population recruited by Balizhuang Community Health Service Center and Dongfeng Community Health Service Center.

The ethics of the study was approved by the Institutional Review Board of Beijing Chaoyang Hospital, Beijing, China (No. 2019-7-24-1). Qualified physicians reviewed electronic medical record systems and approached patients who met inclusion and exclusion criteria. The investigator explained the purpose of the study to the patient who came to the hematology clinic and conducted a face-to-face inquiry after the patient agreed to sign the informed consent form.

### Measures

The demographic characteristics of the participants were collected using an initial questionnaire that included age, gender, race, education level (primary education as low level; secondary school certificate level as medium level; bachelor’s degree or above as high level), and income. In addition, drinking status (over two alcoholic drinks daily on average for more than 1 month was considered drinking; otherwise, no drinking), smoking status (Patients who smoked over 10 cigarettes per day for over 3 months on average was considered smoking; otherwise, no smoking), and body mass index (BMI, kg/m^2^), which is a person’s weight (in kilograms) divided by the square of height (in meters), were also collected. Patients were also investigated whether they had a medical history such as cardiocerebrovascular disease or diabetes mellitus. All participants completed the questionnaire.

### Questionnaires

Patient Health Questionnaire-9 (PHQ-9) (Yu et al., 2012): As a simple and effective self-rating scale, the PHQ-9 is commonly used as an additional diagnostic tool for depression and as a symptom severity assessment tool for outpatients in general hospitals. The internal reliability of the Chinese version of the PHQ-9 was excellent (Cronbach’s α = 0.86) (Wang et al., 2014). The nine items on the PHQ-9, which are rated on a 4-point scale (0 = not at all to 3 = nearly every day) in the previous 2 weeks, are based on depressive symptomatology for the diagnostic criteria in the fourth edition of the Diagnostic and Statistical Manual of Mental Disorders (DSM-IV) in the United States. Depression severity was scored: 0–4 none, 5–9 mild, 10–14 moderate, 15–19 moderately severe, and 20–27 severe.

Generalized Anxiety Disorder 7-item scale (GAD-7) (Cui et al., 2017): The GAD-7, compiled by Spitzer et al. (2006) is a concise self-rating scale for anxiety symptoms according to the diagnostic criteria of the Diagnostic and Statistical Manual of Mental Disorders (DSM-IV). This scale has shown good sensitivity and specificity in screening for anxiety in the Chinese general hospital population. Anxiety severity was scored as follows:0–4 none, 5–9 mild, 10–14 moderate, and 15–21 severe.

Verbal Pain Rating Scale (VPRS) (Chung et al., 1999): There are four pain levels: level 1—painless; level 2—mild (can tolerate, does not affect sleep, can live a normal life); level 3—moderate (obvious pain, disturbed sleep, required general pain relief, sedation, or sleeping pills); and level 4—severe (pain accompanied by autonomic nerve dysfunction and disturbed sleep requiring the use of narcotic drugs).

### Clinical features

Clinical data for *de novo* MM patients were extracted from the database of the hematology department of Beijing Chaoyang Hospital. The baseline clinical parameters comprised: hemoglobin, Hb (g/L); lactate dehydrogenase, LDH (U/l); creatinine, Cr (mol/l); osteolytic lesion; Durie and Salmon (DS) staging system; International Staging System (ISS); Revised International Staging System (R-ISS). Treatment included conventional chemotherapy and novel agent containing therapy. The response was evaluated by the IMWG 2006. Based on the reaction, the MM patients were classified as at least excellent partial response (VGPR) and less than VGPR (Palumbo et al., 2015; Zhang et al., 2017).

### Data analysis

Categorical data was described by counts and proportions. The normal distribution of continuous data was described with means ± standard deviations. The non-normal distribution of continuous data was mainly described by the Median (quartile interval). For the comparison of two groups, independent *t*-tests, rank-sum test, and chi-square tests were used, where appropriate. The rank-based multiple linear regression model was used to estimate the difference in PHQ-9 and GAD-7 between the controls and patients after correcting the relevant variables.

According to the statistical analysis of the case group, the outcome was defined as whether depression or anxiety. The classified data was described by frequency and proportion, and the continuous data was described by mean ± standard deviations. The related factors of depression and anxiety were analyzed, respectively. Chi-square test or *t*-test was used to compare depression and anxiety between the groups. The dependent variables of this study are depression and anxiety in patients with MM. In order to avoid omitting the possible related factors, the factors with *p*-value less than 0.15 were included in multiple logistic regression, and the effects of each clinical indexes on depression and anxiety in patients with MM were analyzed. SAS software version 9.4 was used for data analysis, and two-sided *p*-values of 0.05 were considered statistically significant.

## Results

### Demographic characteristics of patients and controls

183 patients with MM met the criteria, of which 10 refused, and 13 did not adequately complete the survey. From 298 controls in community health centers case-control matched according to sex, age, and BMI (in the ratio of 1:1), 160 patients and 160 controls were involved in the result analysis. Table 1 shows a comparison between patients with MM and controls in terms of sociodemographic characteristics. Compared to the controls, MM patients are more likely to consume more alcohol and have fewer medical conditions. The difference scores in PHQ-9 between the two groups is statistically significant (*Z* = 2.3476, *P* = 0.0189). After adjusting for smoking, drinking, and medical history in rank-based multiple linear regression, there was still a statistical difference in PHQ-9 (*t* = 2.54, *P* = 0.012) between the two groups but was with no significant difference in GAD-7 at the 0.05 test level (*t* = 1.90, *P* = 0.058).

**TABLE 1:** Demographic data and clinical characteristics of MM patients and control subjects.

	Controls	Patients	t/χ^2^/*Z*	*P*
Age, years	57.10 ± 10.93	58.99 ± 10.04	1.61	0.109
Male, n (%)	78 (49.1%)	81 (51.0%)	0.11	0.736
Education, n (%)			3.96	0.138
Low	12 (7.6%)	23 (14.5%)		
Medium	95 (59.7%)	89 (56.0%)		
High	52 (32.7%)	47 (29.5%)		
Drinking, n (%)	17 (10.7%)	39 (24.5%)	10.73	0.001**
Smoking, n (%)	16 (10.1%)	54 (34.0%)	27.63	< 0.001***
Medical history, n (%)	59 (37.1%)	37 (23.3%)	7.27	0.007**
BMI, kg/m^2^	24.59 ± 2.57	24.39 ± 4.02	–0.54	0.588
PHQ-9	2 (0–5)	3 (1–6)	2.3476	0.0189**
GAD-7	1 (0–3)	1 (0–4.25)	1.7347	0.0828

**P < 0.05; ***P < 0.001.

Some subjects did not complete the questionnaire and measures fully, numbers vary slightly in different categories.

### Risk factors associated with depressive symptoms in patients with multiple myeloma

**FIGURE 1:**
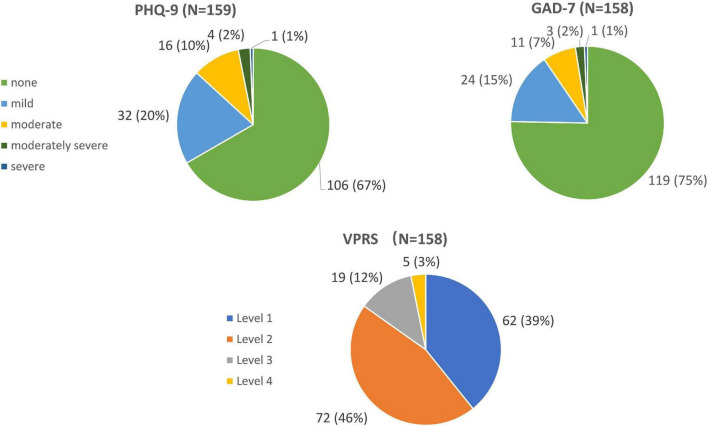
Patients with multiple myeloma during maintenance therapy were scored in PHQ-9, GAD-7, and VPRS.

According to the results of PHQ-9 scores, patients with MM were divided into two groups: no-depression (PHQ < 5) and depression (PHQ ≥ 5). Figure 1 showed patients with MM during maintenance therapy were scored in PHQ-9, GAD-7 and VPRS. Table 2 showed the difference of clinical indexes between depression group and no-depression group. There were statistical differences in BMI, remission state, osteolytic lesion, and pain rating, the threshold was set at *P* < 0.5. Then, these four variables were included in multiple logistic regression analysis. As shown in Table 3, there was a correlation between depression and pain rating. Compared to the pain rating 1, the risk of depressive mood was greater in the case group with the pain rating 2 (OR = 2.38) and the pain rating ≥ 3 (OR = 4.32).

**TABLE 2:** Clinical related factors between depression and no-depression in MM patients.

	Depression *n* (%)	No-depression *n* (%)	χ^2^/*t*	*P*
Gender			0.45	0.501
Female	28 (35.9)	50 (64.1)		
Male	25 (30.9)	56 (69.1)		
* Age	59 ± 9	59 ± 10	0.39	0.697
*BMI kg/m^2^	23.7 ± 3.9	24.7 ± 4.1	–1.52	0.131
Education			0.50	0.779
Low	9 (39.1)	14 (60.9)		
Medium	28 (31.5)	61 (68.5)		
High	16 (34.0)	31 (66.0)		
Drinking			0.00	1.000
No	40 (33.3)	80 (66.7)		
Yes	13 (33.3)	26 (66.7)		
Smoking			0.13	0.722
No	34 (32.4)	71 (67.6)		
Yes	19 (35.2)	35 (64.8)		
Medical history			1.76	0.185
No	44 (36.1)	78 (63.9)		
Yes	9 (24.3)	28 (75.6)		
Hb			0.46	0.499
<100 g/L	26 (36.1)	46 (63.9)		
≥100 g/L	27 (31.0)	60 (67.0)		
LDH			0.11	0.745
<250 U/L	45 (32.9)	92 (67.2)		
≥250 U/L	8 (36.4)	14 (63.6)		
Cr			1.35	0.245
<187 mol/L	46 (35.4)	84 (64.6)		
≥187 mol/L	7 (24.1)	22 (75.9)		
DS stage			1.87	0.393
1	4 (57.1)	3 (42.9)		
2	11 (30.6)	25 (69.4)		
3	38 (33.6)	75 (66.4)		
ISS stage			0.14	0.933
1	11 (35.5)	20 (64.5)		
2	18 (32.1)	38 (67.8)		
3	22 (34.9)	41 (65.1)		
R-ISS 3stage			0.01	0.905
0	41 (35.0)	76 (65.0)		
1	8 (36.4)	14 (63.6)		
Treatment			0.14	0.711
CT	13 (36.1)	23 (63.9)		
NT	40 (32.8)	82 (67.2)		
Remission state			4.28	0.118
VGPR	18 (26.1)	51 (73.9)		
No VGPR	28 (36.8)	48 (63.2)		
PD	6 (54.6)	5 (45.5)		
Osteolytic lesion			3.53	0.060
No	4 (16.7)	20 (83.3)		
Yes	49 (36.3)	86 (63.7)		
Pain			11.34	0.003
1	12 (19.4)	50 (80.7)		
2	28 (38.9)	44 (61.1)		
≥3	13 (54.2)	13 (45.8)		

*Descriptive indicators of continuous data are Mean ± Standard Deviation, statistics are *t*-values.

No depression: PHQ-9 < 5 and depression: PHQ-9 ≥ 5.

Hb, hemoglobin; LDH, lactic dehydrogenase; Cr, creatinine; CT, conventional therapy; NT, novel therapy; VGPR, very good partial remission; PD, progression of disease.

**TABLE 3:** Multiple logistic regression analysis of MM with depression (*n* = 160).

	Parameter estimates	standard error	χ^2^	P	OR	95% confidence interval
Pain-rating (2 vs. 1)	0.87	0.42	4.44	0.035	2.38	1.06–5.33
Pain-rating (3 vs. 1)	1.46	0.53	7.59	0.006	4.32	1.53–12.21
BMI	–0.10	0.05	3.71	0.054	0.91	0.82–1.00
Remission state (No VGPR vs. VGPR)	0.22	0.39	0.33	0.567	1.25	0.58–2.69
Remission state (PD vs. VGPR)	1.18	0.71	2.79	0.095	3.25	0.81–12.98
Osteolytic lesion (Yes vs. No)	0.58	0.62	0.88	0.349	1.78	0.53–5.98

### Risk factors associated with anxiety symptoms in patients with multiple myeloma

According to the results of GAD-7 scores, patients with MM were divided into two groups: no- anxiety (GAD-7 < 5) and anxiety (GAD-7 ≥ 5). Table 4 showed the difference of clinical indexes between no-anxiety group and anxiety group. There were statistical differences in DS stage, remission state, and pain rating, the threshold was set at *P* < 0.5. Then, these three variables were included in multiple logistic regression analysis. The multiple logistic regression showed that there was a correlation between anxiety and pain rating. Patients with a pain level greater than or equal to 3 were at greater risk of anxiety than those with a pain level of 1 (OR = 2.89) (Table 5).

**TABLE 4:** Clinical related factors between anxiety and no-anxiety in MM patients.

	Anxiety *n* (%)	No- anxiety *n* (%)	χ^2^/*t*	*P*
Gender			1.22	0.269
Female	22 (28.6)	55 (71.4)		
Male	17 (21.0)	64 (79.0)		
* Age	59 ± 12	59 ± 10	–0.04	0.967
*BMI kg/m^2^	24.1 ± 3.5	24.5 ± 4.2	–0.63	0.526
Eduction			0.48	0.786
Low	7 (30.4)	16 (69.6)		
Medium	21 (23.6)	68 (76.4)		
High	11 (23.9)	35 (76.1)		
Drinking			0.03	0.873
No	29 (24.4)	90 (75.6)		
Yes	10 (25.6)	29 (74.4)		
Smoking			0.02	0.898
No	26 (25.0)	78 (75.0)		
Yes	13 (24.1)	41 (75.9)		
Medical history			0.69	0.407
No	32 (26.2)	90 (73.8)		
Yes	7 (19.4)	29 (80.6)		
Hb			0.01	0.933
<100 g/L	18 (25.0)	54 (75.0)		
≥100 g/L	21 (24.4)	65 (75.6)		
LDH			0.05	0.819
<250 U/L	34 (25.0)	102 (75.0)		
≥250 U/L	5 (22.7)	17 (77.3)		
Cr			0.01	0.940
<187 mol/L	32 (24.8)	97 (75.2)		
≥187 mol/L	7 (24.1)	22 (75.9)		
DS stage			5.19	0.075
1	4 (57.1)	3 (42.9)		
2	11 (30.6)	25 (69.4)		
3	24 (21.4)	88 (78.6)		
ISS stage			0.01	0.999
1	8 (25.8)	23 (74.2)		
2	14 (25.5)	41 (74.6)		
3	16 (25.4)	47 (74.6)		
R-ISS 3stage			0.10	0.757
0	30 (25.9)	86 (74.1)		
1	5 (22.7)	17 (77.3)		
Treatment			0.02	0.892
CT	9 (25.7)	26 (74.3)		
NT	30 (24.6)	92 (75.4)		
Remission state			4.05	0.132
VGPR	12 (17.4)	57 (82.6)		
No VGPR	24 (31.6)	52 (68.4)		
PD	2 (20.0)	8 (80.0)		
Osteolytic lesion			0.98	0.323
No	4 (16.7)	20 (83.3)		
Yes	35 (26.1)	99 (73.9)		
Pain			6.02	0.035
1	13 (21.0)	49 (79.0)		
2	15 (21.1)	56 (78.9)		
≥3	11 (45.8)	13 (54.2)		

*Descriptive indicators of continuous data are Mean ± Standard Deviation, statistics are *t*-values. No anxiety: GAD-7 < 5 and anxiety: GAD-7 ≥ 5.

Hb, hemoglobin; LDH, lactic dehydrogenase; Cr, creatinine; CT, conventional therapy; NT, novel therapy; VGPR, very good partial remission; PD, progression of disease.

**TABLE 5:** Multiple logistic regression analysis of MM with anxiety (*n* = 160).

	Parameter estimates	standard error	χ^2^	*P*	OR	95% confidence interval
Pain-rating (2 vs. 1)	0.04	0.43	0.01	0.930	1.04	0.44–2.43
Pain-rating (3 vs. 1)	1.06	0.53	4.01	0.040	2.88	1.02–8.15
DS stage (2 vs. 1)	–0.97	0.91	1.15	0.283	0.38	0.06–2.23
DS stage (≥3 vs. 1)	–0.45	0.96	0.22	0.640	0.64	0.10–4.19
Remission state (No VGPR vs. VGPR)	0.74	0.42	3.08	0.079	2.10	0.92–4.82
Remission state (PD vs. VGPR)	0.30	0.89	0.12	0.734	1.35	0.24–7.70

## Discussion

To date, only a few studies have assessed the depressive and anxious states of MM patients in the early survivorship period (Ribeiro-Carvalho et al., 2020; Zaleta et al., 2020). Practitioners generally believe that the anxiety and depression should be eased after maintenance therapy. Therefore, the emotion of patients at maintenance therapy period can often be ignored. However, we noticed that a considerable proportion of the patients existed emotional problems in varying degrees during the maintenance therapy period. Due to the limited clinic data about emotional problems of MM patients and the destitute scientific understanding of factors which affect the mood, the comprehensive and systematic management of the disease is still inadequate; this ultimately leads to poor outcomes. This study focused on the special group of MM during maintenance therapy to explore the correlation between depression and anxiety and the baseline clinical indicators of the disease, so in order to provide scientific evidence for systematic diagnosis, treatment, and psychological support. The results of this study showed that the depressive symptoms’ prevalence rate of MM patients during maintenance is higher than normal people. The higher the patient’s pain rating, the higher the risk of them having depression and anxiety. Factors such as biochemical indicators, disease severity and treatment were not strongly related to the risk of depression and anxiety.

Previous studies have shown that MM is prone to depression (prevalence rate of 26%) and anxiety (prevalence 7.1%) in the early treatment of the disease, which may be due to the lack of certainty of the disease. This increases the psychological burden of patients and predisposing them to acute stress reactions (Oberoi et al., 2017). With the continuous innovation of effective therapeutic agents, the 5-year survival rate of MM exceeds 50% (Alobaidi et al., 2020). One study confirmed that the incidence of depression and anxiety in patients with MM decreases as disease symptoms show improvement. To further elevate the survival rate of this group of patients, reducing the relapse rate is an ongoing goal for practitioners. In recent years, more and more clinicians have begun to focus on the multidisciplinary management of this disease during the maintenance phase. Recent clinical studies have shown that the healthy quality of life and psychological factors of patients with MM are often neglected during the maintenance (Alobaidi et al., 2020). Our study further refined the results of previous studies and found that the prevalence of depressive symptoms in MM during the treatment stage of maintenance reached 33.3%, which was still significantly higher than it in the community population after correcting for other factors. The above study suggests that paying adequate attention to the emotional problems of MM patients during maintenance is crucial to the clinicians.

Pain is the most important factor affecting quality of life in cancer patients (Maindet et al., 2019). According to a literature review study, patients with MM suffer from four common chronic pain at each disease stage: (1) myeloma bone disease-induced pain; (2) chemotherapy-induced peripheral neuro-pain (i.e., bortezomib); (3) post-transplantation immune depression-induced post-herpetic neuralgia; and (4) pain in cancer survivors (Coluzzi et al., 2019). Wilson has demonstrated that the rates of depression in high pain were about two times higher than it in the low pain group (Wilson et al., 2009). A research found that in the complex mechanism of the relationship between pain, anxiety, and depression, neuroplasticity and inflammatory response play an important role (Castellanos et al., 2020). There is correlation between pain arising from PTSD and depression in cancer patients in palliative care (Akechi et al., 2004). The emotional pain suffered by patients have an negative impact on their mental health condition (Singer, 2018). The most crucial finding of our study is that depression and anxiety symptoms are strongly associated with subjective pain levels at the remission phase but not with objective indicators of disease prognosis. Previous study has found that in the treatment of cancer patients, combining psychotherapy with depression and anxiety could significantly improve the quality of life of patients and partially relieve the symptoms of physical pain (Deng, 2019). This study found that with increasing pain level, the risk of depression increased by 2–4 times and the risk of anxiety increased by more than 2 times. Although such patients have achieved significant pain relief during the maintenance therapy period, patients with pain at onset still exhibit symptoms of emotional vulnerability, which may be related to the chronic activation of pain-related inflammation. It According to this study, it suggests that there may be a potential relationship between depression and inflammation.

For patients with cancer pain, clinicians often rely on traditional analgesics to control pain. However, with the prolongation of the action time of analgesics, the risk of drug resistance increases (Zaini et al., 2018). The results of this study showed that depression and anxiety were closely related to pain, which provide an up-to-date research direction for the development of drug targets for pain treatment (Deng, 2019).

## Strengths and limitations

The advantage of this study is the observation of a homogeneous patient group in the clear-cut stage of treatment. The case-control association study obtained a broad understanding of the prevalence of depression and anxiety symptoms with MM patients in China. To the best of our knowledge, this is the first experimental study to analyze clinical characteristics associated with depression and anxiety symptoms in MM. The anxiety and depression of patients during maintenance treatment are not closely related to the objective index of the baseline, but are closely related to pain rating.

On the other hand, there are several methodological limitations in our study. First, due to the limitation of sample size, this study was not involved more factors such as economic conditions, personality, course of disease, and so on (Oliveri et al., 2019). Second, this was a single-center study and MM patients was diagnosed and treated in Beijing Chaoyang Hospital. Multicenter clinical trials will be further needed to follow all stages of the disease, and explored a bio-psycho-social intervention model to guide patients’ treatment.

## Conclusion

The results showed that excluding acute stress response at the initial stage of the disease, MM patients still had a certain proportion of anxiety and depression when they were in the “functional cure” stage. The anxiety and depression of patients during maintenance are not closely related to objective indicators of the baseline, but to pain. Depression and anxiety symptoms are strongly associated with subjective pain levels at the remission phase but not with objective indicators of disease prognosis. Under the multi-disciplinary management mode of diagnosis and treatment, it is important to focus on emotional problems in patients with MM during maintenance stage of treatment. Physicians should regularly evaluate MM patients’ state of depression and anxiety, especially paying attention to the patients who have complained of pain at the time of onset. Because of these investigations, it is proved that a psycho-biological integrated intervention model is essential to guide patients’ treatment comprehensively, systematically and scientifically.

## Data availability statement

The raw data supporting the conclusions of this article will be made available by the authors, without undue reservation.

## Ethics statement

Ethical approval was obtained from the Ethics Committee of the Medical Faculty of Beijing Chao-Yang Hospital, Capital Medical University (Approval Date: July 24; No. 2019-7-24-1). The patients/participants provided their written informed consent to participate in this study. Written informed consent was obtained from the individual(s) for the publication of any potentially identifiable images or data included in this article.

## Author contributions

HS: conceptualization, formal analysis, methodology, writing—original draft and review and editing, and supervision. HR: methodology, original draft—review and editing, and data curation. YT: conceptualization, methodology, and data curation. ZC: data curation, methodology, and project administration. CX, LL, XM, and XL: original draft, methodology, and data curation. WC: conceptualization and supervision. TG: supervision and methodology. CF and WG: conceptualization, supervision, methodology, and original draft—review and editing. All authors contributed to the article and approved the submitted version.
